# Enhancing the interfacial binding strength between modular stretchable electronic components

**DOI:** 10.1093/nsr/nwac172

**Published:** 2022-08-29

**Authors:** Shaobo Ji, Xiaodong Chen

**Affiliations:** Innovative Centre for Flexible Devices (iFLEX), Max Planck-NTU Joint Lab for Artificial Senses, School of Materials Science and Engineering, Nanyang Technological University, Singapore 639798, Singapore; Innovative Centre for Flexible Devices (iFLEX), Max Planck-NTU Joint Lab for Artificial Senses, School of Materials Science and Engineering, Nanyang Technological University, Singapore 639798, Singapore; Institute of Materials Research and Engineering (IMRE), Agency for Science Technology and Research, Singapore 138634, Singapore

**Keywords:** stretchable electronics, interfacial strength, covalent bonding, mechanical interlocking, device–human interfaces

## Abstract

Stretchable electronics are emerging for personalized and decentralized clinics, wearable devices and human–machine interactions. Nowadays, separated stretchable functional parts have been well developed and are approaching practical usage. However, the production of whole stretchable devices with full functions still faces a huge challenge: the integration of different components, which was hindered by the mechanical mismatch and stress/strain concentration at the connection interfaces. To avoid connection failure in stretchable devices, a new research focus is to improve the interfacial binding strength between different components. In this review, recent developments to enhance interfacial strength in wearable/implantable electronics are introduced and catalogued into three major strategies: (i) covalent bonding between different device parts, (ii) molecular interpenetration or mechanical interlocking at the interfaces and (iii) covalent connection between the human body and devices. Besides reviewing current methods, we also discuss the existing challenges and possible improvements for stretchable devices from the aspect of interfacial connections.

## Teaser

This review discusses the covalent bonding and physical interlocking strategies to enhance the interfacial binding strength between different stretchable electronic components, and proposes prospects of building stretchable interconnects.

## INTRODUCTION

Smart devices for decentralized and personalized clinics and sports management have become quite common in our daily life. Beyond well-developed portable electrocardiography (ECG) recorders, blood pressure and sugar-level detectors, etc., commercial wearable devices that can continuously monitor biomarkers in real time are also emerging, which are based on mature rigid or flexible polyimide (PI) circuit boards. However, unstretchable systems do not perform well for wearable or implantable biomarker collection, especially for continuous monitoring during body motion, due to the mismatched mechanical properties between signal collection units (e.g. electrodes, chemical sensors, optical sensors) and subjects (e.g. skin, tissues). Their mismatch results in distinct deformation of collectors and subjects, leading to unstable interfaces and thus signal drifting, motion artifacts and even signal loss. This drives the development of stretchable sensors and devices that can conformally deform with the substrates for wearable and implantable applications [1–5].

Stretchable electronics can retain their properties and functions during deformation by endowing stretchable substrates with electrical functionalities. Similar to conventional rigid electronics, various stretchable components have been developed for different purposes, such as stretchable conductors, sensors, power sources, etc. Stretchable conductors and energy storage (batteries and supercapacitors) have provided the foundation for integrated stretchable devices [6–13]. Stretchable electrodes and sensors guarantee their functions by conformally adhering to skin and tissues for stable biomarker collections including electrophysiological signals, pulse, respiration rate, temperature, etc. [14,15]. The continuous real-time signals provide not only more information for healthcare but also accurate feedback for human–machine interactions [16–18]. Stretchable electronics can interact with humans more reliably and comfortably compared to traditional electronics, representing the future for wearable devices.

In the early stage of stretchable electronics, the focus was to design and fabricate stretchable functional components. After years of research, the separated functional parts have been well developed and are approaching practical applications. With more functions and miniaturized stretchable components, a new challenge arose in the production of intact stretchable devices from lab to commercialization: the connection and integration of different stretchable and rigid electronic components. The combination of different units will result in mechanical mismatch and/or low binding strength at the connection interfaces. Compared to stretchable electronics, this may not be a serious issue in commercial rigid and PI-based flexible devices. They are fabricated in the form of printed circuit boards and the connections are realized by soldering or mechanical fixation, providing stable interfaces. Besides, they undergo limited deformation (bending to developable surfaces) compared to stretchable ones (deform to non-developable surfaces with heterogeneous stretching) [19], which further lowers the influence of the mismatch. However, it is crucial for stretchable electronics as, compared to flexible ones, the mismatch will lead to higher stress/strain concentration at the hybrid interconnect points when being stretched [20,21] and the weaker interface will eventually lead to the failure of the whole device.

**Figure 1.**
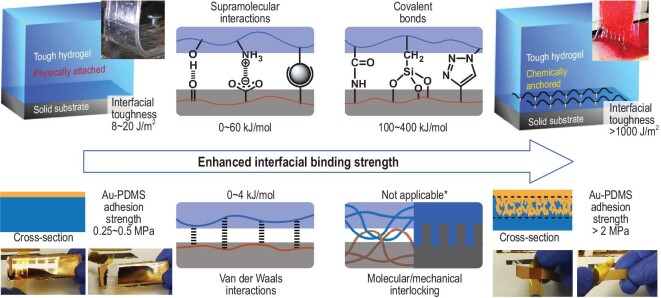
General strategies for enhancing interfacial binding strength in stretchable electronics. Above: introducing high-bond-energy covalent bonds to replace low-bond-energy supramolecular interactions. Adapted with permission from Ref. [23], Copyright 2015, Nature Publishing Group. Below: introducing interlocking structure with complication interactions to replace weak van der Waals interactions. Adapted with permission from Ref. [25], Copyright 2019, WILEY-VCH Verlag GmbH & Co. KGaA, Weinheim. *Cannot be denoted as kJ/mol as it is highly dependent on materials and interlocking structures.

To solve this problem, one of the strategies is to enhance the interfacial binding strength between different functional components or materials, thus overriding the property and mechanical mismatch at the connection points. Popular methods include building covalent bonds instead of non-covalent interactions at the interfaces and constructing molecular or mechanical interlock structures (Fig. 1). Covalent bonds have much higher bond energy than non-covalent supramolecular interactions that play significant roles in adhesive systems (Fig. 1, above) [22]; for example, by covalently anchoring a hydrogel layer to a solid substrate using amide bonds, their interfacial toughness could be improved from <20 J/m^2^ (physically attached, hydrogen bonds between carboxyl groups in hydrogel and oxides in solid) to >1000 J/m^2^ [23], showing the great promise of the covalent binding strategy. Meanwhile, not all materials are suitable to form covalent bonds and interlocking structures are their alternatives. Compared to simple van der Waals interactions, interlocking structures could introduce more complicated interactions, including molecular/mechanical entanglements, vertical/horizontal frictions and enlarged contact areas, thus enhancing the overall interfacial strength (Fig. 1, below) [24]. For instance, it is difficult to build covalent bonds at the interface between a deposited gold nanolayer and an elastic PDMS substrate, resulting in low Au–PDMS adhesion strength. By purposely forming interlocking structures, the adhesion strength could be improved from 0.25∼0.5 to >2 MPa, producing much more stable stretchable conductors and electrodes [25].

In this review, we have focused on the above-mentioned two strategies to introduce recent developments and summarize the major methods for enhancing the interfacial binding strength between different stretchable components. In the field of flexible electronics, such strategies were not only applied in device fabrication for improved interlayer binding, but also used to produce robust device–human interfaces, especially between electrodes and skin/tissues. These contents will be discussed in three parts: (i) interfacial covalent bonding between different device parts, (ii) molecular interpenetration or mechanical interlocking between different device parts and (iii) covalent connection between the human body and devices. Besides reviewing current methods, we also discuss the existing challenges, possible improvements and prospects for the production of stretchable devices from the aspect of interfacial connections.

### Covalent bonds enhance device interfaces

In manufacturing, the combination of different components is commonly realized by welding and adhesives. Stretchable electronics are based on polymers and elastomers, and are thus not suitable for the conventional welding process so adhesion between different units is the major way of integrating different parts. Adhesion is mainly achieved through forming non-covalent interactions, such as hydrogen bonds, coordination bonds, van der Waals and electrostatic interactions, etc. [26–28]. They could provide reversible adhesion and, with optimization, relatively high interfacial strength. Such interactions have been studied to drive the assembly and binding of macro-sized materials, with the potential for smart and reversible manufacturing of soft materials [29–32]. However, in stretchable electronics, the connection between different parts frequently undergoes stress/strain concentration and requires more robust binding. Facing such challenges, researchers have developed covalent bond-based interfacial binding. The covalent bonds have much higher bond energy compared to non-covalent ones and can provide higher interfacial binding strength, though the reversibility is compromised.

For serpentine-structured and PI-based stretchable electronics, the overall packaging has been realized by stepwise curing of silicone rubbers, during which these encapsulation layers are easily and covalently connected [33–36] whereas for multifunctional and intrinsically stretchable electronics, the devices are expected to be fabricated from various materials and functional layers to fulfill complicated tasks. It requires the combination of substrates with different mechanical and chemical properties, such as rigid electronics units/circuits and stretchable electrodes/sensors. Their covalent connections can only be achieved through purposeful design and processing. After years of research, two major strategies for the formation of covalent bonds to enhance the interfacial strength have been developed: grafting or growing another component on the surface via *in situ* polymerization of monomers; and connecting two parts via the reaction between the two surfaces. They will be discussed with typical examples.

### Grafting/growing another component

**Figure 2.**
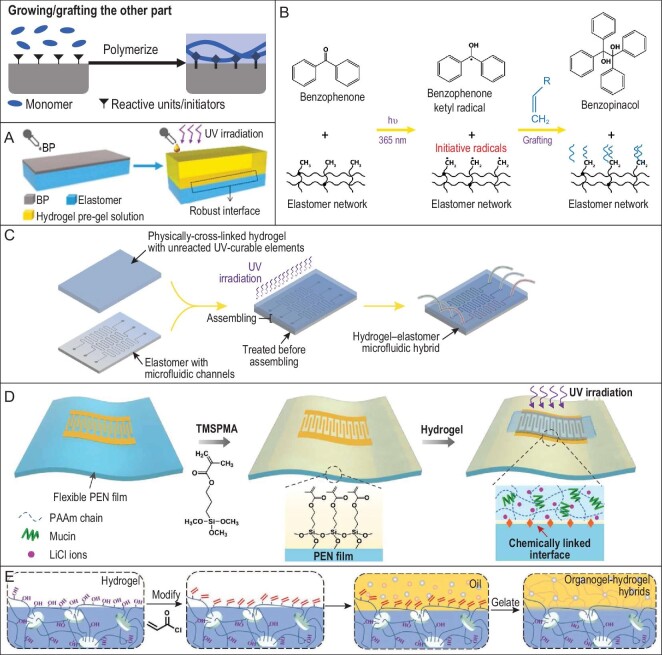
Covalently grafting/growing another component via initiators. (A) Benzophenone (BP) as a photo initiator to covalently bind hydrogels with PDMS elastomers. PDMS functioned as the electrification layer and hydrogel as the electrode to produce a triboelectric nanogenerator. Adapted with permission from Ref. [40], Copyright 2018, American Chemical Society. (B) The initiation mechanism of BP swelled elastomers. Adapted with permission from Ref. [41]. (C) BP-initiated covalent linking between pre-gel and microstructured elastomers to produce robust hybrid microfluidics and stretchable hydrogel circuit boards patterned on elastomers. Adapted with permission from Ref. [41]. (D) Surface grafting of reactive monomers to covalently bond hydrogel sensor on flexible electrodes through co-polymerization. Adapted with permission from Ref. [43]. (E) Grafting monomers on hydrogel surfaces via ester bonds for covalently bonding between organogels and hydrogels. Adapted with permission from Ref. [44], Copyright 2018, WILEY-VCH Verlag GmbH & Co. KGaA, Weinheim.

The first discussed strategy, grafting/growing another layer, is usually used for tough binding between hydrogels and substrates due to their special properties, functions and fabrications. Hydrogels have played irreplaceable roles in stretchable electronics especially in biosignal collections, due to their biocompatibility, ionic conductivity and tissue-level modulus [37–39]. Thus, it is important to realize the tough binding of hydrogel components on stretchable substrates to produce stable and reliable flexible devices. The adhesion energy of hydrogels on other substrates is determined by both the toughness of the hydrogel and the interfacial binding strength. Here we only focus on the methods to improve the interfacial binding strength (the intrinsic adhesion energy), as they are expected to be applicable for materials more than hydrogels. The issue with hydrogels is that they contain a large amount of water, which makes them hydrophilic with a lowered polymer chain density that leads to decreased non-covalent interaction sites on their surfaces. Together they result in a low affinity of hydrogels to common substrates. To overcome this problem, researchers have taken advantage of the fabrication of acrylate hydrogels—that is, they can be produced from the radical polymerization of monomers. By introducing reactive sites on substrate surfaces, the polymer scaffolds in hydrogels can be covalently linked to the substrate during the *in situ* polymerization and formation of the gels (Fig. 2).

The reactive sites could be initiators or monomers. Benzophenone (BP) is the most-used initiator for such applications. For example, a triboelectric nanogenerator with PDMS as the electrification layer and hydrogel as the electrode was reported with tough interfacial binding (Fig. 2A) [40]. The PDMS elastomer was swollen with BP, which triggers the gelation of hydrogel pre-solution and covalent connection between the PDMS and polymers in the gel under UV irradiation. The ions in the hydrogel and the tough interfaces ensured the stable mechanical and electrical performances of the devices. The reaction mechanism is shown in Fig. 2B [41]; under UV light, BP transformed the methyl group in the PDMS to initiative radicals that induced the polymerization of monomers in the hydrogel pre-solution and covalently grew the gel on the PDMS surface. Such a strategy was further used to combine physically cross-linked pre-gel with elastic substrates (Fig. 2C) [41]. The advantage of using pre-gel over pre-solution is the possibility to keep hollow structures between the two layers. Robust hydrogel–elastomer hybrid microfluidics and stretchable hydrogel circuit boards patterned on elastomers were fabricated in such a way with enhanced interfacial toughness (>1500 J/m^2^ compared to 3.5 J/m^2^ without covalent bonding). By designing the microstructures and incubating microbes in the hydrogel–elastomer hybrids, stretchable living devices have also been fabricated with chemical sensing ability [42].

BP-initiated connection is easy to operate, but only applicable to limited substrates that can swell in the BP solution and contain hydrocarbons to form initiative radicals. Meanwhile, although it requires more steps, surface modification of reactive units could greatly broaden the applicable substrates for hydrogel grafting with little influence on their mechanical properties. In such systems, the substrates first undergo a covalent modification step to grow a monolayer of units that could react with groups in the hydrogel. The units are usually acrylates that could be polymerized with the monomers in the gel to form covalent connections. For example, a hydrogel artificial tongue was produced by covalently anchoring a hydrogel sensor on poly (ethylene naphthalate) (PEN)-based electrodes (Fig. 2D) [43]. The PEN film was first modified using a layer of 3-(trimethoxysilyl)propyl methacrylate (TMSPMA) and then the methacrylates on the PEN surface were polymerized into the hydrogel to provide covalent bonds at the interface. The tough interface between the chemical sensing hydrogel and electrodes resulted in the stable performance of the artificial tongues. The hydrogel could be not only the grafting layer, but also the grafted substrate. By introducing hydroxyl groups to the polymer chain in the hydrogel, its surface could be modified to acrylates through esterification (Fig. 2E) [44]. Then an organogel layer was covalently grown on the hydrogel by the co-polymerization of the hydrophobic monomers and the surface acrylates. It provided a method to protect the hydrogel from drying and swelling, enhancing the stability and elongating the lifetime of functional hydrogels.

**Figure 3.**
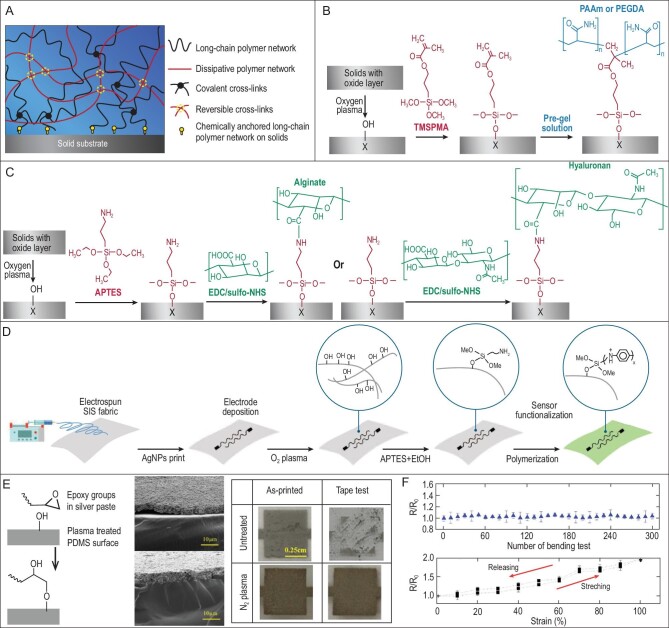
Covalently grafting/growing another component via reactive groups. (A) A universal method to produce tough binding of hydrogels on various solid substrates. The polymer networks were covalently anchored through (B) co-polymerization or (C) EDC-Sulfo NHS-facilitated amide bond formation. Adapted with permission from Ref. [23], Copyright 2015, Nature Publishing Group. (D) Surface-modified amine groups could co-polymerize with aniline to covalently link stretchable substrate with conductive polyaniline (PANI). It produced stretchable electronics sensors from commonly rigid PANI. Adapted with permission from Ref. [51], Copyright 2021, Wiley-VCH GmbH. (E) The reaction between epoxy in the silver paste and plasma-treated PDMS surface resulted in robust interfaces. (F) The stability and performance of silver-paste-coated PDMS as stretchable conductors. Adapted with permission from Ref. [52], Copyright 2016, American Chemical Society.

Based on the surface modification and tough hydrogels, a universal method was proposed to realize the tough bonding of hydrogels on various solid substrates (Fig. 3A) [23]. The solid surfaces were plasma-treated to form hydroxyl groups and then modified with reactive units through siloxane covalent chemistry. Different reactive units were utilized for different gels. Surface-modified methylacrylates could co-polymerize with acrylate monomers or cross-linkers to covalently anchor hydrogels on solid surfaces from pre-gel solutions (Fig. 3B). As acrylates are very common monomers for hydrogel fabrication, the surface methylacrylate-based method is suitable for a wide range of gels with varying mechanical properties and is easy to realize whereas surface-modified amines could graft gels with carboxyl groups through EDC-Sulfo *N*-hydroxysuccinimide (NHS) chemistry (Fig. 3C). It could covalently anchor hydrogels formed by bio-macromolecules such as alginate and hyaluronan, the biocompatible and degradable gels being more suitable for applications requiring the substrates to be worn and implanted. The surface amine-based method could also be realized after gel formation for building 3D structured gel surfaces. The robust covalent anchoring at the hydrogel–substrate interfaces and the high toughness of the gel resulted in interfacial toughness of >1000 J/m^2^ for various solid substrates (8–20 J/m^2^ without covalent bonds). This universal method could be used to graft hydrogels onto flexible substrates and electrodes to improve the performance and stability of stretchable devices or in implantable medical devices to enhance their lubricity and biocompatibility [45].

Besides the integration with hydrogels, another important issue in stretchable electronics is the binding of conductive materials with stretchable substrates. However, most conductive materials used lack adequate reactive units, such as metals and carbon materials [46–50]. Their enhanced interfacial strength is usually achieved via mechanical methods (see section ‘Nano to macro interlocking enhanced interfaces’) and only limited conductive materials have been covalently grafted onto substrates. One of the examples benefited from the *in situ* polymerization of a conducting polymer, polyaniline (PANI) (Fig. 3D) [51]. The substrate polystyrene-*b*-polyisoprene-*b*-polystyrene (SIS) fabric was modified with a layer of amine groups, which could polymerize with aniline to covalently graft the PANI onto the fabrics. PANI itself is a rigid material, but the covalent bonding with the soft substrate endowed the whole sensor with stretchability and stable electrical and sensing performances. Another example is to covalently link commercial silver paste with PDMS substrates (Fig. 3E) [52]. One of the major components in silver paste is epoxy resin and their epoxy groups could directly react with hydroxyl groups on the plasma-treated PDMS surface. The SEM images revealed an obvious difference between untreated and treated samples, and the tape test exhibited distinct interfacial strength. The tough interface resulted in better mechanical electrical performance as stretchable conductors (Fig. 3F).

The grafting/growing strategy is facile for stretchable systems involving hydrogels and materials with adequate reactive units. And it can provide tough interfacial binding regardless of the different surface properties of the linked components. However, in current research, this strategy is too specialized for gels and always requires *in situ* formation of one layer from monomers. It on the one hand restricted applicable materials and on the other hand limited the possible fabrication methods, especially for devices with 2D or 3D structures. For this strategy, the future research focus should be to develop more functions for gel systems so that they can replace other materials to overcome the restriction. While broadening the applicable materials, the following strategy might be more suitable: connecting two parts via reactions at their interface.

### Bridging two parts via chemical reactions

**Figure 4.**
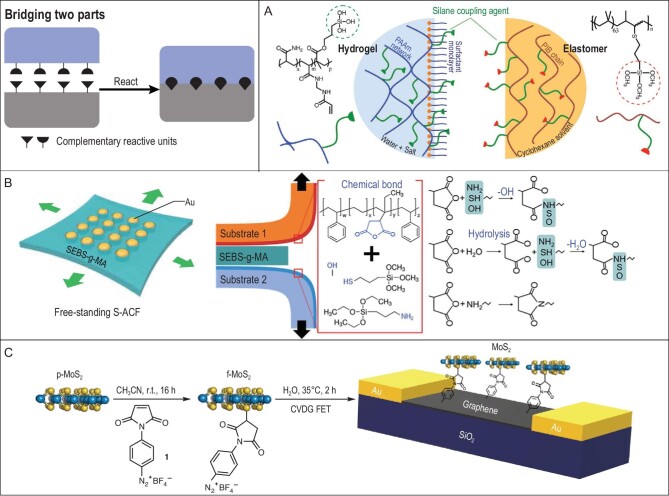
Bridging two components via chemical interactions. (A) A textile conductor from a covalently linked hydrogel core and elastomer sheath. The protective cover makes the conductor washable without swelling the gel core. Adapted with permission from Ref. [53], Copyright 2017, American Chemical Society. (B) A stretchable anisotropic conductive (ACF) film that could covalently connect two substrates with high resolution of ≤50 μm. Adapted with permission from Ref. [54]. (C) Covalently linked functional 2D–2D heterostructures from MoS_2_ and graphene. Adapted with permission from Ref. [59], Copyright 2022, The Author(s), under exclusive license to Springer Nature Limited.

Compared to grafting or growing another part on the surface, directly connecting two components has fewer limitations on the used materials. Most materials can be surface-modified with specific reactive units and, ideally, different parts could be covalently integrated through their reactions (Fig. 4). The disadvantage of this strategy over the grafting/growing method is mainly from the interfacial contact: when the surface of two solid parts attach to each other, the mismatch of surface morphology could result in a lowered contact area and thus reduced interfacial strength. For example, in a macroscopic supramolecular assembly system, the PDMS cubes could hardly form intact contact at the nanometer scale, resulting in the formation of little supramolecular interactions and the failure of the cube assembly [29]. By adding softer spacing coating layers on the surfaces via layer-by-layer assembly, the surfaces could deform to match each other, thus increasing the formation of supramolecular interactions and enhancing the apparent coupling constant to facilitate the cube assembly, whereas in the field of stretchable electronics, this issue could be mostly addressed by using soft substrates (such as hydrogels and rubbers that are much softer than PDMS) and/or smooth pristine surfaces.

Hydrogels are usually soft and can have good contact with other substrates, and the gel is required to be filled in wires or tubes in some applications, such as stretchable conductive wires or surface modification of artificial blood vessels for improved biocompatibility. Under such circumstances, the gel needs to grow toward the inside and the direct bonding between a cured gel core and the outer covering is more convenient. A flexible textile conductor was fabricated with a salted hydrogel core and an elastomer sheath [53]. Reactive trimethoxysilane groups were purposely grafted onto the polymer network of the hydrogel and elastomer (Fig. 4A). To form an intact sealed cover, the elastomer was used in the form of a solution instead of a bulk film; the conductor was produced by dip coating the hydrogel fiber in the elastomer solution and further drying the solvents. The siloxane provided enhanced interfacial toughness (79.3 ± 13.9 J/m^2^) compared to without covalent bonds (2.37 ± 0.04 J/m^2^) and the sheath made the conductor fibers washable without obvious swelling or drying of the functional hydrogel core.

Due to the high interfacial strength and universality, such a strategy is playing a more and more important role in flexible electronics. Commercial anisotropic conductive films (ACFs) are widely used in the connection of rigid electronic units that avoids the risk of short circuits. However, in stretchable electronics, the adhesion strength of ACFs is not enough to sustain the deformation at the connection points. To address this issue, covalent bonds were proposed to replace the non-covalent interaction-based adhesion in commercial ACFs [54]. As shown in Fig. 4B, gold microparticles were embedded in stretchable substrates, producing an anisotropic conductivity along the vertical direction. This stretchable-ACF (S-ACF) and the substrates to be connected were all modified with reactive groups. The S-ACF contains maleic anhydride and the substrate surfaces were grafted with hydroxyl, thiol or amine groups. The S-ACF could react with the surfaces under pressure (0.1 MPa for intact contact, 80^o^C to facilitate the reactions) to produce enhanced interfacial connection with a resolution of 50 μm and a stretchability of 70% biaxial strain.

Besides macro-level connection, with the development of flexible electronics and the smaller size of investigated subjects and devices, the molecular- and nano-level connection is also becoming important to improve the stability and performance of stretchable electronics, especially the field-effect transistor (FET) arrays. Building stretchable FET arrays with miniaturized size is crucial for fabricating flexible chips and sensors. Covalent bond-improved binding has also contributed to their development. At the molecular level, UV-triggered carbene insertion cross-linking has been recently introduced to flexible electronics (Fig. 4C) [55,56]. It has been used to crosslink a polymer matrix, conducting polymers and polymer semiconductors for high-density optical microlithography of elastic circuits and transistor arrays. It has also been used to covalently link different functional layers in the FET and resulted in enhanced peel strength between the polymer semiconductor and flexible substrate (>112 compared to 57 N/m without covalent bonds) [57]. The advantage of carbene insertion cross-linking is that it can be used as long as a C–H bond exists in the substrates to be connected. At the nano level, the covalent connection has been demonstrated to improve the performance of 2D material-based devices by cross-linking the MoS_2_ flakes in the functional layer [58]. Furthermore, functional 2D–2D heterostructures by covalently linking MoS_2_ and graphene were fabricated (Fig. 4D) [59], which were usually based on van der Waals interactions [60].

Beyond the above-mentioned covalent reactions, a cold-welding derived method was also used in flexible electronics to provide improved connection. Direct gold bonding could be considered as ‘metal bonds’ instead of covalent bonds [61]. After water vapor plasma treatment, the gold surfaces were cleaned and could form seamless contact with each other. The gold bonding resulted in robust interfaces with little voids; there might also be hydroxyl groups and covalent reactions involved in this process. In this way, the connection part could have similar strength as to an ACF connected, but with much higher flexibility (conformal to curvatures with a radius of 0.5 mm and for ACF connection it is 5 mm).

Covalent bonds can significantly enhance the interfacial strength and connection points in stretchable electronics, making them more reliable and repeatable. Though the resulting performance is improved, the covalent strategy is more complicated than non-covalent methods (adhesives). It always requires stepwise modification and reaction, and the reactive units each have their selectivity toward substrates or complementary units. The universal UV-triggered carbene insertion cross-linking is a rare example that requires no modification but only one reaction step, with relatively high universality. But it is still of high cost [3-phenyl-3-(trifluoromethyl)-3H-diazirin group containing cross-linker] and requires UV irradiation that could only be used in transparent parts. The ideal covalent connection method should be universal, low-cost, controllable and highly efficient under mild conditions. Developing such methods is an important direction for the research of enhancing the interfacial strength in flexible devices and crucial for the fabrication and mass production of robust and reliable commercial devices.

### Mechanical interlocking enhanced device interfaces

The formation of covalent bonds can improve the connection and interfacial strength, yet it still has some requirements for the connected components: the substrate materials must possess certain activity for covalent bonding and the function of the materials should not be affected by the chemical reactions. However, there are several widely used functional materials in flexible electronics that cannot fulfill both the requirements, such as relatively inert gold and some conducting polymers that depend on intact conjugated structures [46,62,63]. For such materials, another strategy is proposed: construct mechanical interlocking structures at the interfaces.

Mechanical interlocking structures can enlarge the interfacial contact area to enhance the van der Waals force between two surfaces. It can also induce additional forces at the interfaces: chain entanglement for molecular-level interlocking, friction force and mechanical force for nano- to macro-level interlocking. The enlarged contact area and additional forces from mechanical interlocking also result in enhanced interfacial strength. Moreover, this strategy was not used only for the above-mentioned materials, but also for gels through polymer network interpenetration. In this section, the mechanical interlocking strategy will be exampled and discussed on two different levels: molecular interpenetration and nano- to macro-level interlocking structures.

### Molecular interpenetration enhanced interfaces

Most stretchable components are polymers and, besides covalent bonds, polymer chain entanglement is another important force to endow the materials with mechanical strength. From this aspect, the chain entanglement between different components—that is, molecular interpenetration—will also help to enhance their interfacial binding strength. This is particularly suitable for gels, as they have a relatively loose network compared to the dense bulk polymer materials, which allows easier formation of interpenetrated networks. When the networks are fully overlapped, it will be a double-network gel [64,65], while when the networks only overlap at the edges, it will be an enhanced interface connection.

**Figure 5.**
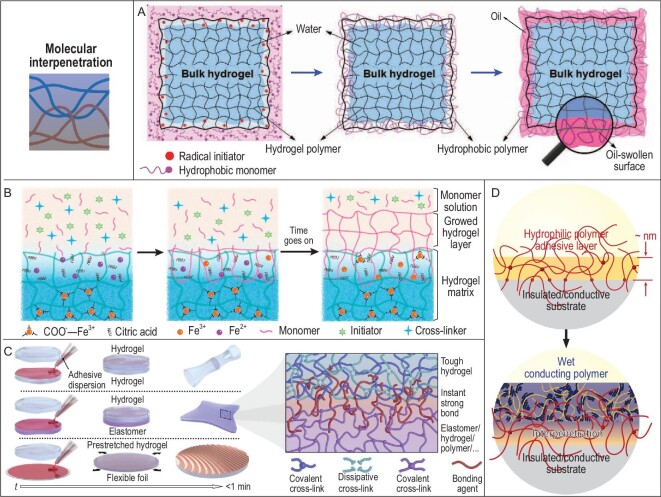
Molecular interpenetration at the interfaces to enhance the binding. (A) The growing of an organogel layer covering a hydrogel through the formation of interpenetrated polymer networks. The organogel layer protected the hydrogel to maintain mechanical properties and functions. Adapted with permission from Ref. [66]. (B) The process of growing additional hydrogel layers via Fe^2+^ catalysed initiation. Adapted with permission from Ref. [67], Copyright 2021, Elsevier Inc. (C) Instant bonding of hydrogels on various substrates via the formation of interpenetrating networks from the adhesive dispersion. Adapted with permission from Ref. [69]. (D) Robust binding of wet conducting polymers on polymer adhesive layer buffered substrates. Adapted with permission from Ref. [73].

The interpenetration of polymer networks has been used to add layers in hydrogel systems. For example, a hydrogel with initiators swollen at the surface was immersed in hydrophobic monomers; the polymerization was limited at the hydrogel surface and resulted in an interpenetrated hydrophobic layer (Fig. 5A) [66]. The layer turned into an organogel after swelling oil and protected the hydrogel from drying or swelling to keep its mechanical properties and functions. In another study, the hydrogel surface was swollen with iron ions as the catalyst for initiation and when in contact with a pre-gel solution, a new hydrogel layer could be grown with a seamless interlocked interface (Fig. 5B) [67]. The swelling and growth could be repeated and the new layer could be controlled by catalyst concentration, producing multi-layer hydrogels and gradient hydrogels. This method could also be used to coat functional hydrogel layers on various solid substrates [68]. It is quite useful in the fabrication of multifunctional hydrogels for stretchable electronics. The bonding of cured hydrogels on various substrates has also been realized through molecular interpenetration (Fig. 5C) [69]. The interpenetrating network was not realized from gel monomers; instead, it was built by an adhesive dispersion that was composed of cyanoacrylate monomers and non-solvent alkanes. The dispersion allowed the adhesive monomers to diffuse into the hydrogel and other substrates, and polymerize to form physically entangled networks at the interface, providing enhanced bonding with interfacial toughness of >2000 J/m^2^. The diffusion also avoided the formation of rigid resin interlayers from cyanoacrylates, thus keeping the stretchability of the substrates. This method has been used to fabricate various stretchable devices, including soft adaptive lenses, stretchable energy harvesters and batteries, and stretchable circuits, showing its great potential in stretchable electronics. In these examples, unlike BP initiation, the polymerizations were restricted to reactive monomers and would not react with existing polymer chains, thus no covalent connection was formed and no damage to the original substrate would be induced.

Molecular interpenetration was also used to bind conducting polymers on electrode substrates. Poly(3,4-ethylenedioxythiophene):poly(styrene sulfonate) (PEDOT:PSS) and polypyrrole (PPy) are promising conducting polymers for flexible electronics due to their biocompatibility [70,71]. But they are usually rigid and must be tightly bound to stretchable substrates to avoid fracture or delamination during deformations. Covalent grafting may only anchor part of the polymers and influence the conjugated structures and electrical properties. To address this problem, especially during application in wet environments such as on sweaty skin or contacting tissues, molecular interpenetration methods were proposed. PPy has been interfacially polymerized on silk fibroin (SF) layers to produce stretchable electrodes [72]. The SF layer contained Fe^3+^ ions that could initiate the polymerization of pyrrole and, through the polymerization at the interface between SF and pyrrole solution, an interlocking structure was formed. PPy is rigid with poor stretchability, but the interlocking layer enhanced the PPy/SF interfacial adhesion strength (1.91 compared to 0.71 MPa if directly pasting the layers) and unified the stretchability of PPy and SF, resulting in a stretchable electrode (300% maximum strain compared to 1% without interlocking). The PPy produced conductivity for signal collection, while the SF layer produced enhanced adhesion after sweating, which was confirmed by the adhesion energy at high relative humidity that simulated sweating. A universal binding method for wet conducting polymers was also reported as shown in Fig. 5D; hydrophilic polymer adhesive layer that was a few nanometers thick was introduced to substrates via covalent modification [73]. This layer functioned as a buffering layer to produce polymer chain interpenetration with conducting polymers and resulted in enhanced adhesion. The lap-shear strength by the wet conducting polymer reached 110–160 kPa on various substrates, while it was only 0.1 kPa on pristine glass. In this way, conducting polymers were tightly deposited on diverse electrodes for implanted monitoring of various electrophysiological signals.

Molecular interpenetration is quite suitable for gels and polymer-based materials; the entangled polymer chains could provide enhanced interfacial strength to improve the connections between two polymer-based parts. However, it is not suitable for materials aside from polymers, such as metals and carbon materials. Moreover, it usually requires manipulation during the formation of the polymers and is thus not suitable for already fabricated bulk materials. For such systems, the interlocking has to be at the nano, micro or even macro level rather than molecular level.

### Nano to macro interlocking enhanced interfaces

Beyond polymer chains, nanomaterials, nanostructures and micro/macro structures are also frequently encountered components in flexible electronics. In such systems, it is difficult to build up interlocking at the molecular level and covalent bonds may not be easy to be introduced to the interfaces either, such as for metals. Under these circumstances, larger-scale mechanical interlocking can greatly enhance the interfaces and connections.

**Figure 6.**
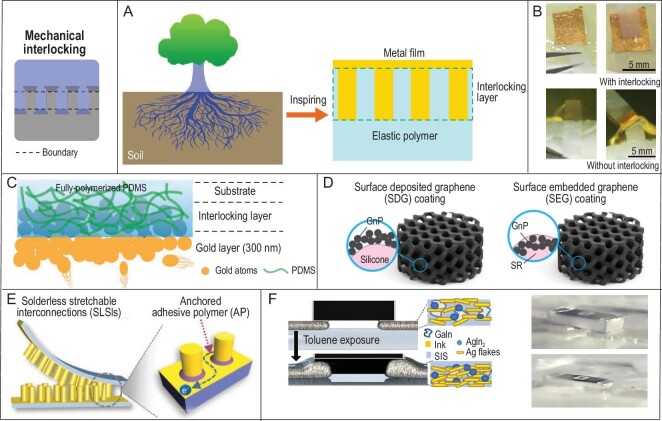
Nano-, micro- and macro-level mechanical interlocking at the interfaces. (A) Inspiration from tree roots to interlocking structure enhanced interfaces. (B) Au/PDMS electrodes with interlocking structures stayed intact after tape peeling, while gold was removed by tape from normal Au/PDMS. Adapted with permission from Ref. [74], Copyright 2016, WILEY-VCH Verlag GmbH & Co. KGaA, Weinheim. (C) Thermal-radiation-assisted gold encapsulation in PDMS. Adapted with permission from Ref. [25], Copyright 2019, WILEY-VCH Verlag GmbH & Co. KGaA, Weinheim. (D) By locking the embedded graphene nanoplatelets (GnPs) in silicone rubber, the produced sensors had a much-elongated shelf life and high stability. Adapted with permission from Ref. [79], Copyright 2020, American Chemical Society. (E) Illustration of micropillar-based interlocking for stretchable interconnects with adhesive polymer as assistance. It resulted in high stretchability compared to conventional solder methods. Adapted with permission from Ref. [80], Copyright 2022, Wiley-VCH GmbH. (F) Styrene–isoprene block copolymers (SIS)-based interlocked soft–rigid hybrid circuits. After toluene exposure, the ink and rigid units would sink into the SIS gel to form macro interlocking structures. Adapted with permission from Ref. [81].

Tree roots in nature have inspired the design and fabrication of interlocking structure enhanced Au/PDMS interfaces (Fig. 6A) [74]. Gold nanolayer deposited elastomers, especially PDMS, are one of the most popular stretchable conductors and electrodes in stretchable electronics [75–77]. But the Au/PDMS interfaces are quite weak; the gold can be easily removed from the substrate by tapes, rubbing or friction, even with the help of a Cr buffering layer. Inspired by tree roots, nanopiles were introduced into the gold layer as ‘roots’ to enhance the interfacial strength with the PDMS ‘soil’. The interlocking resulted in more robust adhesion of the gold onto the PDMS so that the gold stayed on the PDMS after tape peeling, while the gold was easily removed by tape for Au/PDMS without interlocking structures (Fig. 6B). The nanopiles were fabricated from nano-templates and the process was complicated with limited productivity. To overcome this problem, the fabrication method was further optimized to thermal-radiation-assisted gold encapsulation in the PDMS [25]. The illustrative result is shown in Fig. 6C: semi-cured PDMS was used as the substrate for gold evaporation and the gold atoms could diffuse into the uncured PDMS. During this process, the PDMS would be gradually cured due to the heat from the gold source and atoms, while the gold would be encapsulated in the PDMS and form connected nano-interlocking structures with the cured PDMS. This method significantly reduced the complexity of producing Au/PDMS with robust interfaces and the reverse sides were also black rather than golden, indicating the formation of nanostructures. These two methods have enhanced the Au/PDMS interfacial adhesion strength from 0.25∼0.5 to 2.0∼2.6 MPa, and the electrical stretchability from 10% to 130% strain (without the Cr layer). By controlling the conditions, such interlocking nanostructures could also be formed during the metal deposition on some plastic rubbers, such as SEBS [78].

Carbon materials-based flexible electronics have also benefited from interlocking structures. A flexible sensor was produced from silicone rubber and graphene nanoplatelets (GnPs) [79]. As GnPs were not in the form of a bulk film, the interlocking was realized not by interfaces, but by directly locking the embedded GnPs onto the surface of silicone rubbers (Fig. 6D). The locking significantly increased the stability of the sensors compared to that with simple surface deposited GnPs. They had a longer shelf life and were more stable toward solvent treatment. This study demonstrated that even simple mechanical embedding could to some extent improve the performance of flexible devices.

As mechanical interlocking is a straightforward and relatively simple method, it also works well on a larger scale. For example, micro interlocking structures have been applied to build robust interconnects in stretchable electronics (Fig. 6E) [80]. Micropillars were constructed as bridges for the connection with adhesive polymer as assistance. The pillars provided an additional contact area and mechanical force to enhance the connection and conductive paths for electrons to travel between the connected parts. Compared to connections based on conventional solders that would cause stress/strain concentration, the solderless micro interlocking strategy could sustain much higher sheer strain (35% over 5%). It provides a robust connection method for stretchable electronics. Furthermore, mechanical locking has also been used on a macro scale to provide enhanced connection in stretchable electronics. A styrene–isoprene block copolymers (SIS)-based system was developed for soft–rigid hybrid stretchable circuits (Fig. 6F) [81]. In the circuit fabrication, a stretchable conductive ink from SIS, silver flakes and liquid metal was first printed on the SIS substrate, then conventional rigid electronics units, such as chips, were placed on the conductive ink patterns. The prepared hybrid circuits were further exposed to toluene vapor to trigger the polymer–gel transition of the SIS substrate, and the inks and rigid units would sink into the soft gel state SIS. After drying, the circuits were interlocked to the SIS substrate with enhanced interfacial strength and overall stability. The polymer–gel transition-induced interlocking structure could improve the conductivity of the ink (from 3.8 × 10^5^ to 8.2 × 10^5^ S/m) and the chip-integrated hybrid circuits could sustain tensile strains of >500%.

Mechanical interlocking is a convenient way to improve interfacial strength. It is suitable for more types of materials compared to the covalent bonding strategy as it only requires mechanical properties. The interfacial toughness significantly depends on the property of the materials and the interlocking structure, which on the one hand means uncertainty and on the other hand a broader tunable range. By controlling the structures, it is also possible to fabricate reversible yet robust connections. However, molecular interpenetration is only applicable to swollen polymers or gels so further research is needed to broaden the applicable substrates, such as co-polymerization methods for different bulk elastomers, or methods to facilitate the diffusion and chain entanglement at the interfaces. Macro interlocking structures are the easiest ones to fabricate with the development of advanced manufacturing methods; for example, the interlocking structures could be designed and directly 3D printed for usage. But the micro, especially nano, interlocking structures are still complicated to fabricate. They usually need specially fabricated molds to generate the structures and careful demolding is required to avoid damaging the small-scale structures. For the mechanical interlocking enhanced interfaces, an important direction is the development of precise manufacturing methods, such as 3D micro/nano printing and 3D lithography that can directly produce desired interlocking structures during the fabrication of stretchable components. The realization of batch processing of micro/nano-interlocking structures will remarkably drive the combination and connection of various stretchable electronic units, and even lead to Lego-like individualized integration of personal devices.

### Covalent bonds enhanced device–skin/tissue interfaces

The most important and irreplaceable role of stretchable electronics is wearable or implantable bio-monitoring devices. Depending on the applications, some devices do not require long-lasting and stable device–human interfaces, such as optical sensors, static ECG (usually 10-s recording time in clinic) or short-term electromyography. In such scenarios, weak and repeatable bonding of sensors or devices with skin is more desirable as it is convenient, comfortable and materials-saving. However, current stretchable wearable devices are targeting long-term, continuous and reliable monitoring of biosignals in daily life for the next generation of smart healthcare systems. They are desired to deform with the human body or tissues during routine activities to keep an intact and stable device–human interface for a long time (hours to days), thus collecting reliable signals with minimum artifacts; in such cases, strong bonding is preferred. Mechanical mismatch and surface property differences still exist between the stretchable devices and biological tissues [82]. During deformation or after long-term wearing, current stretchable devices still face the challenges of unstable interfaces, especially for electrodes that detect electrophysiological signals. A minor change in the electrode–substrate interface will result in a major draft and artifact in the recorded signal. It is still important to develop methods to build robust device–human interfaces, especially for electrodes.

**Figure 7.**
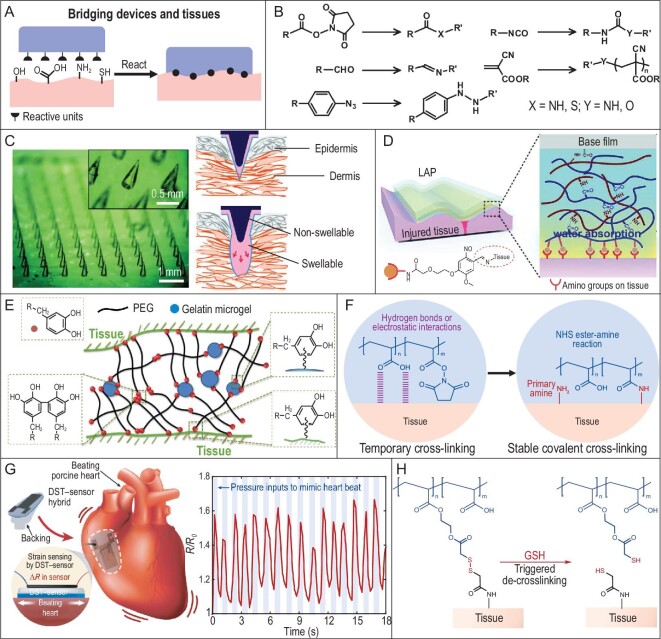
Enhancing device–human interfaces. (A) Reactive groups on skin and tissue surfaces. (B) Reactions between components from medical adhesives and tissue surface groups. (C) Microneedle arrays to construct mechanical interlocking structures between device and skin. Adapted with permission from Ref. [84], Copyright 2013, Nature Publishing Group. (D) Tissue adhesive patches based on covalent Schiff base formation with tissue surface amine groups. Adapted with permission from Ref. [88]. (E) Dopamine adhesive and gelatin microgel-based tissue adhesives. The dopamine groups could covalently bond to tissue surfaces to enhance the adhesion. Adapted with permission from Ref. [90]. (F) The reaction between *N*-hydroxysuccinimide (NHS) ester and skin/tissue surface amine groups for building stable covalent cross-linking. Adapted with permission from Ref. [91], Copyright 2019, The Author(s), under exclusive license to Springer Nature Limited. (G) A covalently fixed strain sensor on the porcine heart that could detect simulated heartbeats. Adapted with permission from Ref. [91], Copyright 2019, The Author(s), under exclusive license to Springer Nature Limited. (H) The introduction of disulfide bonds could endow the covalently enhanced interfaces with responsive benign detachment. Adapted with permission from Ref. [93].

The human skin is protected with a corneum layer, while the tissues are in a wet environment. In both cases, common adhesives do not function well. But the surface chemical groups, mainly hydroxyl and amine groups (Fig. 7A) from biomolecules such as transmembrane proteins and glycoproteins, provide an opportunity for additional anchoring forces on the skin and tissues [24]. Medical glues have been developed based on covalent reactions with these surface groups. Some typical reactions involved are shown in Fig. 7B [83]. Though they provide high adhesion to skin and tissues, the medical glues have potential toxicity (acrylates, aldehydes) due to their high reactivity and, after reaction, the glues become rigid, leading to restriction or deformation. Thus, conventional medical glues could not solve the device–human interface problems, from the aspects of safety, comfort and conformal deformation. But the reactions involved have inspired the enhancement of device–human interfaces via covalent bonds.

Mechanical interlocking has rarely been used for the purpose of enhancing interfacial strength in the human body. As skin and tissues have intact surfaces, the formation of interlocking structures will inevitably damage them—that is, in an invasive way. In one of the limited examples, microneedle arrays were used to induce mechanical interlocking with skin (Fig. 7C) [84]. The outer layer of the microneedles would swell body fluid and expand after penetrating the epidermis to form interlocking structures. This design improved the adhesion of flexible patches on the skin, as the adhesion strength increased from 0.28 ± 0.11 to 0.93 ± 0.23 N/cm^2^ with microneedles and the work of adhesion from 0.6 ± 0.13 to 5.23 ± 1.7 mJ/cm^2^. It provided the possibility to use mechanical interlocking to improve device–human interfaces, but the invasive microneedles would also cause tissue deformation and open wounds and thus it is not a good choice when considering comfort, long-term usage and possible infections. Due to the invasive nature, microneedles are more suitable and mostly reported for applications that require direct contact with body fluids, such as drug delivery, chemical sensing and electrodes for accurate under-skin signal conductions [85–87].

Because of the inconvenience of mechanically interlocking wearable devices and the human body, covalent bonding is currently the most effective method to build robust device–human interfaces. There are several covalent bonds used for making skin and tissue adhesive patches. For example, an adhesive patch could be light-activated to covalently bond to tissues via photo conversion from o-nitrobenzene to o-nitrosobenzaldehyde groups and subsequent formation of Schiff base with amine groups on tissue surfaces (Fig. 7D) [88]. Dopamine groups have also been utilized to covalently bond to tissues [89,90]. An injectable adhesive was fabricated by combining gelatin microgels and dopamine-based adhesives (Fig. 7E). The microgels improved the curing rate and mechanical properties of the adhesive networks, while the dopamine groups could covalently bond to tissues for enhanced interfacial adhesion.

Recently, the formation of amide bonds from activated NHS ester and skin/tissue surface amine groups became one of the most popular covalent adhesion strategies. A dry-tape adhesion system for wet tissues and devices was reported (Fig. 7F) [91]. The dry tapes would swell the surface water to facilitate the contact with tissue surfaces and the reaction formed covalent amide bonds to provide robust interfacial binding. Together they resulted in fast and tough tissue adhesion with interfacial toughness of >710 J/m^2^ (<20 J/m^2^ for commercial bioadhesives) and shear strength of >120 kPa (<45 kPa for commercial bioadhesives). The tapes could not only repair and seal damaged tissues, but also fix flexible devices onto tissues. A strain sensor was fixed onto a porcine heart and could detect the simulated heartbeats (Fig. 7G). A multifunctional origami patch has also been fabricated from the tapes benefitting from their dry and paper-like properties [92]. The patch could seal tissues with a hydrophobic layer to drive away body fluids and an anti-bacterial layer to prevent further infection. The chemical structures of the tapes have also been varied according to requirements. As shown in Fig. 7H, the introduction of disulfide bonds at specific positions led to responsive benign detachment of adhesive patches or devices from tissues [93].

**Figure 8.**
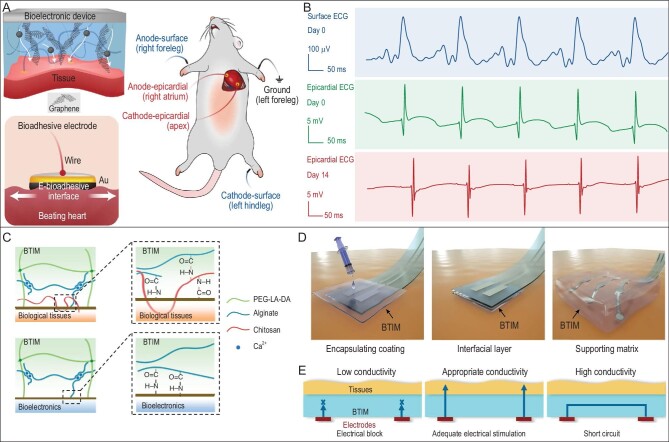
Covalent bonds enhanced stretchable electrode–tissues interfaces. (A) The addition of graphene endowed the covalently anchored adhesive with conductivity and could fix electrodes on the tissue to collect electrophysiological signals. (B) The covalently anchored electrodes on the heart could record stable epicardial ECG for 14 days after implantation. Adapted with permission from Ref. [94], Copyright 2020, The Author(s), under exclusive license to Springer Nature Limited. (C) Bioresorbable adhesives for building covalent connections at the device–tissue interfaces. (D) The solution and pre-gel adhesives could be used in various forms. (E) The crosstalk among neighboring electrodes could be avoided by controlling the gel thickness and conductivity. Adapted with permission from Ref. [95], Copyright 2021, The Author(s), under exclusive license to Springer Nature Limited.

In flexible electronics, stretchable electrodes benefited the most from the covalent bonds enhanced tough device–human interfaces. By adding a conductive filler, graphene, into the dry tape, an electrical bioadhesive was produced (Fig. 8A) [94]. The covalent bonds constructed a robust interface to keep the constant electrode–tissue contact even in wet environments, while the graphene provided conductivity to transfer the electrophysiological signals. The tough electrode–heart interface (interfacial toughness >420 J/m^2^) guaranteed stable epicardial ECG recording for ≥14 days of implantation. In other research, the adhesives for electrodes were used in another way [95]. Alginate, calcium ions and PEG-based macromonomers were mixed to form a pre-gel. Under UV light, the macromonomer was cross-linked to form the final gels. In the presence of a primer that contains carbodiimide and Sulfo NHS, the carboxyl groups in alginate were activated to react with the amine groups on the tissue surface and covalently bind the gels onto the tissues (Fig. 8C). The PEG-based macromonomers contained polylactic acid segments and made the gel bioresorbable. The form of solution and pre-gel expanded the possible way of using the adhesives; they could be used to cover the device, bridge the device–tissue interface or as the supporting matrix of electrodes (Fig. 8D). In this research, crosstalk among neighboring electrodes was avoided by controlling the conductivity and gel thickness (Fig. 8E), which greatly simplified the fabrication of adhesive electrode arrays.

Research on covalent-bond-enhanced device–human interfaces has provided an excellent candidate particularly for building tough electrode–tissue interfaces; other types of devices currently have a much lower requirement for stable interfaces. Another popular strategy is to use ultra-thin or nanomesh electrodes that are fully compliant with soft tissues [96,97]. They could provide feelingless monitoring and represent one direction for future wearable and implantable biosignal collection devices, even though such devices are relatively difficult to operate. Meanwhile, being fully compliant does not mean that ultra-thin/nanomesh electrodes have high interfacial toughness; they still face adhesion problems. The combination of covalent interfaces and ultra-thin electrodes could be a promising direction for realizing practical bio-monitoring devices. From the aspect of bioadhesives, to fulfill requirements for wearable and implantable devices, research should push forward the development of the following properties: stretchable after the adhesive cures that would not form rigid layers as acrylate adhesives; tunable degradability for biosorption of adhesives and devices targeting different lifetimes; reversible and responsive transformations between adhesive and non-adhesive for controllable detachment and reuse; reactions that provide fast and specific cross-linking but with low toxicity; injectable single-component adhesives for convenient applications; and ability to form ultra-thin adhesion layers to collaborate with ultra-thin devices.

## CONCLUSION AND PERSPECTIVES

Interfaces and connections play an important role in the fabrication and application of stretchable electronics, as they either integrate different components to make whole functional devices or bridge substrates and devices to transfer biosignals. However, they are also always the weakest points during deformation due to mechanical mismatch and stress/strain concentration. Enhancing the interfacial binding strength and interconnects between different stretchable electronic components is one of the research focuses to push forward the applications and productions of stretchable electronics. With years of research, several strategies have been proposed and developed, in both chemical and physical ways (quantitative data from the discussed examples are summarized in Table 1). Researchers have used mechanical interlocking structures and high-bond-energy covalent bonds (10–400 kJ/mol) to replace the low-bond-energy non-covalent interactions (0–60 kJ/mol) and van der Waals interactions (0–4 kJ/mol) that account for the binding strength in common adhesives. Chemically, through polymerization of monomers in the presence of reactive components, the new part could be covalently anchored or molecularly interlocked with the substrates. Two different components could also be covalently connected via the reactions between surface groups, thus tightly integrating devices or binding devices on the human body regardless of the surface property and mechanical mismatch. Physically, molecular interpenetration was used to connect different polymer networks, especially in hydrogel materials. And larger-scale mechanical interlocking has been studied for the cases without the contact of different polymers. Nano interlocking provided stretchable devices with better performance, micro interlocking was used to build stable stretchable connections and macro interlocking was applied to produce stretchable circuits.

**Table 1.** Summarized quantitative data from the discussed examples.

Component 1	Component 2	Method	Improvement measurement, value (control)	Ref.
Hydrogel	Elastomer	Chemical bonding	Interfacial toughness, >1500 J/m^2^(3.5 J/m^2^)	[41]
Hydrogel	Elastomer	Chemical bonding	Interfacial toughness, 79.3 ± 13.9 J/m^2^ (2.37 ± 0.04 J/m^2^)	[53]
Hydrogel	Solid	Chemical bonding	Interfacial toughness, >1000 J/m^2^(8–20 J/m^2^)	[23]
PDPPT-TT	PET	Chemical bonding	Peel strength, >112 N/m (57 N/m)	[57]
Double-sided tape	Skin	Chemical bonding	Interfacial toughness, >710 J/m^2^ (<20 J/m^2^)Shear strength, >120 kPa (<45 kPa)	[91]
Electrodes	Epicardium	Chemical bonding	Interfacial toughness, >420 J/m^2^ (1.55 J/m^2^)Shear strength, >110 kPa (0.21 kPa)	[94]
Electrodes	Skin	Chemical bonding	Adhesion energy, 300 ± 70 J/m^2^	[95]
Electrodes	Epicardium	Chemical bonding	Adhesion energy, 240 ± 20 J/m^2^	[95]
Au	PDMS	Mechanical interlocking	Adhesion strength, 2.0 MPa (0.5 MPa)Electrical stretchability, >130% (<10%)	[25]
Au	PDMS	Mechanical interlocking	Adhesion strength, 2.6 MPa (0.25 MPa)	[74]
Au/PDMS	Au/PDMS	Mechanical interlocking	Interconnect stretchability, 35% (<5%)	[80]
Rigid unit	SEBS circuit	Mechanical interlocking	Chip-integrated circuit stretchability, >500%	[81]
Flexible patch	Skin	Mechanical interlocking	Adhesion strength, 0.93 ± 0.23 N/cm^2^ (0.28 ± 0.11 N/cm^2^)Work of adhesion, 5.23 ± 1.7 mJ/cm^2^ (0.6 ± 0.13 mJ/cm^2^)	[84]
PPy	Silk fibroin	Molecular interpenetration	Adhesion strength, 1.91 MPa (0.71 MPa)Overall stretchability, >300% (<0.1%)	[72]
PEDOT:PSS	Solid	Molecular interpenetration	Lap-shear strength, 110–160 kPa(0.1 kPa)	[73]

These strategies have solved some problems in interfaces and connections, yet still have a long way to go toward ideal stretchable electronics. First, the methods should be simpler to lower the cost and facilitate device production. The chemical way still requires several steps of modification and the physical way usually requires delicate templates, especially at the nano and micro levels. New methods should be developed, for example, universal and low-cost reactive cross-linkers that could react with various pristine material surfaces, nano and micro 3D printing or lithography methods for constructing mechanical structures. Second, covalent binding to conventional electronics components should be studied. The covalent fixation of rigid components onto stretchable circuits, instead of solder, adhesives or liquid metals, might produce robust rigid–soft hybrid circuits and is worth being researched. Third, universal binding and connection systems should be developed for the standardized production of stretchable electronics. Current methods usually require the pairing of complementary parts or from the same/similar materials. Modular assembly of stretchable electronics has been achieved by using self-healing polymers as the major matrix [98], yet the limited choice of materials could hardly fulfill complicated functions in commercialized wearable devices. A general system is desired to produce stretchable and rigid parts with universal connections so that they could be tightly connected between any two parts regardless of their materials and properties. It could pave the way for ‘Lego-like’ assembly of modularized stretchable devices from different functional blocks, which suits their desired applications: personalized and customized functions with massive production. And finally, reversible yet strong connections should be studied based on dynamic covalent bonds. The introduction of disulfide bonds to device–human interfaces has resulted in tough but detachable adhesion [93]; such a strategy could be used in modularized devices for controlled and facile disassembly/reassembly of the functional blocks to better reuse/recycle the electronics toward a sustainable future. Some dynamic bonds have specific responsiveness and have the potential to introduce additional features to the devices, such as visible-light-responsive diselenide bonds that might provide better controllability to the block assembly benefitting from the high spatial resolution of visible light [99,100].

In brief, there are still a lot of challenges in the interfacial connection of stretchable electronics and overcoming them is crucial for the production and commercialization of stretchable wearable devices. The development and optimization of covalent and mechanical interlocking methods have promoted and will continue to promote to overcome the challenges.
